# Oxidative stress mediated oleanolic acid therapeutic potential in rheumatoid arthritis: focus on the mechanisms

**DOI:** 10.3389/fphar.2026.1741004

**Published:** 2026-04-30

**Authors:** Xinyu Lai, Junhua He, Anyi Liao, Jian Feng, Li Deng

**Affiliations:** 1 School of Clinical Medicine, Chengdu University of Traditional Chinese Medicine, Chengdu, China; 2 Department of Cardiology, The Affiliated Hospital of Southwest Medical University, Southwest Medical University, Luzhou, China; 3 Traffic Accident Prevention and Handling Brigade, Traffic Management Detachment, Chengdu Municipal Public Security Bureau, Chengdu, China; 4 Department of Rheumatology, The Affiliated Hospital of Southwest Medical University, Luzhou, China

**Keywords:** NF-κB, NOX, Nrf2, oleanolic acid, oxidative stress, REAGE, rheumatoid arthritis

## Abstract

Rheumatoid arthritis (RA) is a chronic systemic autoimmune disease of unknown etiology. Several signaling pathways associated with oxidative stress, such as Nrf2, NOX and NF-κB, have been implicated in the pathogenesis of RA. Promoted reactive oxygen species cause the damage of lipids, nucleic acid and proteins, leading to RA. Given the evidence of the damage caused by oxidative stress to rheumatoid arthritis, the use of antioxidants has become a comprehensive approach for treating the disease. The natural antioxidant oleanolic acid (OA) modulates several signaling pathways connected to redox imbalance, thereby mitigating oxidative injury. Medical research has confirmed that OA has significant therapeutic potential in a variety of diseases through anti-oxidation, anti-inflammation, anti-apoptosis, and participates in the regulation of glucose and lipid metabolism. This article reviews the signaling pathways and antioxidant activity of OA in the pathogenesis of RA, as well as the research progress in the prevention and treatment of RA in recent years, to identify therapeutic targets and new antioxidant treatment methods for RA.

## Introduction

Rheumatoid arthritis (RA) is a global health challenge that poses a considerable threat to human life, and its epidemiological characteristics and clinical hazards have attracted considerable attention. It is estimated that 0.5%–1% of the global population is affected by RA, and there is a significant sex difference (female: male = 3:1) (Phull et al., 2018). In terms of disease progression, approximately 80% of patients develop varying degrees of dysfunction within 20 years after diagnosis, and 20%–30% of patients without standardized treatment develop irreversible disability within 2–3 years after the onset of the disease (Rindfleisch and Muller, 2005; Schmajuk et al., 2011). RA is a chronic systemic autoimmune disease of unknown etiology. In addition to the typical joint symptoms (persistent pain, swelling, and morning stiffness), RA is often accompanied by multi-system involvement, including extra-articular manifestations such as subcutaneous nodule formation, interstitial lung disease, and vasculitis, and systemic complications such as vascular system abnormalities and metabolic disorders.

In the pathological process of RA, pro-inflammatory cytokines (such as tumor necrosis factor superfamily members) and oxidative stress are deeply intertwined, each amplifying the other in a self-sustaining cycle (Vinay and Kwon, 2012; Rasheed and Haqqi, 2008). Oxidative stress (OS) is fundamentally defined as a dynamic imbalance between the generation rate of reactive oxygen species (ROS) and the antioxidant defense capacity of cells. And this state induces oxidative modification of biological macromolecules (DNA, lipids, and proteins), further aggravating the progression of RA (Phull et al., 2018). In general, OS is characterized by a dynamic imbalance between the generation rate of reactive oxygen species (ROS) and the antioxidant defense capacity of cells. In RA, this redox imbalance is not merely an epiphenomenon but a core pathological driver: excess ROS directly damage synovial tissue, modify self-antigens to trigger autoimmune responses, and activate downstream inflammatory pathways that perpetuate joint destruction (Phull et al., 2018). Based on the above mechanisms, targeted scavenging of ROS or restoration of homeostasis of the oxidation-antioxidant system has become an important research direction in current RA treatment strategies.

**FIGURE 1:**
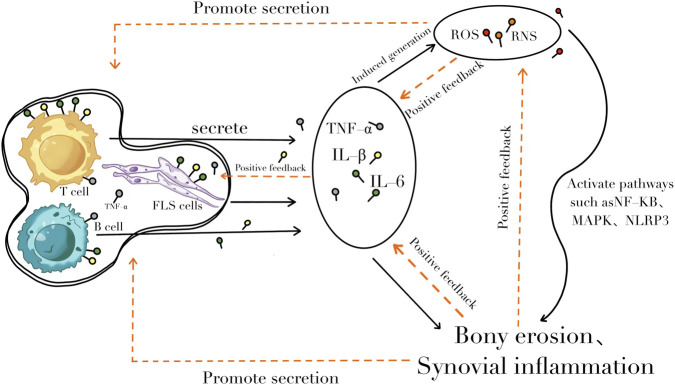
The pathophysiological mechanism of rheumatoid arthritis. The occurrence and development of rheumatoid arthritis involve multiple mechanisms, including activation of T/B cells, oxidative stress, cytokine storm, and formation of synovitis.

In the search for better therapeutic strategies, researchers have turned to widely available and safe extracts of natural products. Therefore, the research on natural products of RA has gradually increased in recent years, and oleanolic acid (OA), as one of them, has attracted wide attention. OA is a pentacyclic triterpenoid metabolite that is widely distributed throughout the plant kingdom. This class of metabolites and their derivatives have shown a variety of pharmacological properties with broad application prospects in nature, including liver protection, inflammation inhibition, free radical scavenging and tumor growth intervention, which have been widely recognized (Pollier and Goossens, 2012; Wang et al., 2010; Lin et al., 2016). Medical research has confirmed that OA has significant therapeutic potential in a variety of diseases through anti-oxidation, anti-inflammation, anti-apoptosis, and other effects and participates in the regulation of glucose and lipid metabolism (Lin et al., 2016). This article reviews the signaling pathways and antioxidant activity of OA in the pathogenesis of RA, as well as the research progress in the prevention and treatment of RA in recent years, to identify therapeutic targets and new antioxidant treatment methods for RA (Figure 1).

## Search strategy and study selection

To ensure a comprehensive and systematic review of the pharmacological effects of OA on RA, a literature search was conducted across multiple electronic databases, including PubMed, Scopus, Web of Science, and the Cochrane Library. Search Strings and Databases the search spanned articles published from inception up to January 2026, with a specific emphasis on high-quality peer-reviewed studies published between 2023 and 2025 to ensure the inclusion of the most recent advancements. The search strategy utilized combinations of the following Medical Subject Headings (MeSH) terms and keywords: Compound-related: “Oleanolic acid,” “3β-hydroxy-olea-12-en-28-oic acid,” “pentacyclic triterpenoids.” Disease-related: “Rheumatoid Arthritis,” “RA,” “synovial fibroblasts,” “collagen-induced arthritis.” Mechanism-related: “Oxidative stress,” “Nrf2 pathway,” “NF-κB signaling,”, “mitochondrial dysfunction,” “AGE/RAGE axis.” Boolean operators (AND, OR) were applied to refine the results (e.g., “Oleanolic acid” AND “Rheumatoid Arthritis” AND “Nrf2”). All botanical sources mentioned in this review were cross-referenced and validated through the Medicinal Plant Names Services (MPNS) and Plants of the World Online (POWO). Inclusion and Exclusion Criteria Studies were included if they met the following criteria: Original research investigating the antioxidant or anti-inflammatory effects of OA specifically in RA models (*in vitro*, *in vivo*, or clinical). Studies exploring the molecular mechanisms involving redox-sensitive pathways. Peer-reviewed articles published in English. Exclusion criteria included: Studies focusing on unrelated triterpenoids without a direct comparison to OA.

## Role of oxidative stress in RA

Oxidative stress plays a key role in the pathophysiology of RA. Clinical controlled studies (n = 120 RA patients vs. 53 healthy controls) have shown that RA patients have a significant increase in the generation of reactive oxygen species (ROS), which manifests as the accumulation of lipid peroxidation products, protein oxidation, and DNA damage in the serum and synovial fluid, suggesting a systemic oxidative stress state, which further aggravates the chronic process of RA (Mateen et al., 2016). Such oxidative damage may also promote the production of rheumatoid factor by inducing autoantigen modification (e.g., IgG oxidation), thereby accelerating joint tissue degeneration (Spreng et al., 2001; Uesugi et al., 2000).

Longitudinal epidemiological studies have further revealed the association between antioxidant status and RA: Analysis of a cohort of 1,419 Finnish adults showed that low antioxidant status appeared to be a risk factor for initiating and developing RA over a follow-up period of up to 20 years. An elevated risk of RA was also observed with lower β-carotene and selenium levels (Heliövaara et al., 1994). A dietary survey of 29,368 women confirmed that the intake of antioxidant vitamins and trace elements is inversely related to the incidence of RA (Cerhan et al., 2003). In addition, the co-existence of glutathione depletion and elevated lipid peroxidation markers (such as MDA) in the plasma and red blood cells of RA patients suggests a multiway disorder of the antioxidant defense network, which also reflects that RA patients are more susceptible to free radical-mediated oxidative damage (Vijayakumar et al., 2006). At the cellular level, the following cells are involved in the oxidative stress process in the context of RA. Modern pathological studies focus on the central role of fibroblast-like synoviocytes (FLS) in the synovial microenvironment. As the predominant cells in the synovial lining, activated FLS produce large amounts of ROS through upregulated NOX4 expression, promoting VCAM1 and VEGF expression to enhance their migration and invasion (Lee et al., 2020). These cells receive and amplify RA-specific signals such as TNF-α. Simultaneously, the inflammatory environment triggers macrophages to polarize predominantly toward the pro-inflammatory M1 phenotype, which significantly outweighs the anti-inflammatory M2 phenotype in affected areas (Lee et al., 2020). While M2 cells secrete IL-10 to alleviate inflammation, M1 cells release TNF-β and IL-6, aggravating the disease.

Furthermore, neutrophils undergo oxidative stress-driven NETosis, releasing citrullinated histones that act as autoantigens to activate T cells (Carmona-Rivera et al., 2024). Once overactivated, T cell subsets (such as Th17) secrete cytokines like IL-17, IL-1β, and IL-6, alongside chemokines (CXCL1, CXCL2, CXCL8), which further recruit neutrophils and exacerbate the local oxidative environment (Ding et al., 2024). By releasing RANKL to drive osteoclastogenesis, these activated T cells translate oxidative stress-induced immune dysfunction into persistent joint bone erosion.

At the molecular level, oxidative stress aggravates the progression of RA by causing biomacromolecule damage, which induces the depolymerization of hyaluronic acid and reduces joint lubrication function (Gringhuis et al., 2000). Abnormal immune regulation: Oxidative modified hyaluronic acid inhibits T cell response and weakens immune homeostasis (Gringhuis et al., 2000); Promoting the release of pro-inflammatory mediators: the level of nitric oxide (NO) in the synovial fluid of RA patients is significantly higher than that in healthy people (Mateen et al., 2016), and excessive NO stress aggravates inflammation. While clinical and experimental data collectively suggest that oxidative stress is a significant biomarker of RA severity, its role as a definitive pathological driver in human patients remains a subject of intense debate. Although ROS participate in the inflammatory cascade, translating ROS-targeted interventions into clinical benefits has proven exceptionally challenging. Unlike the robust results in animal models, the clinical evidence that ROS regulation can fundamentally alter the human disease course is limited and often contradictory (Sies and Jones, 2020).

## Oxidative stress signaling pathways in rheumatoid arthritis

Several signaling pathways associated with oxidative stress—including nuclear factor erythroid 2-related factor 2 (Nrf2), advanced glycation end products (AGEs), nicotinamide adenine dinucleotide phosphate (NADPH) oxidase (NOX), and nuclear factor kappa light-chain enhancer of activated B cells (NF-κB)—have been implicated in the pathogenesis of RA (Xu et al., 2023).

### Nrf2 signaling

Nrf2 (Nuclear factor erythroid 2-related factor 2) belongs to the basic leucine zip (bZIP) transcription factor family, and its members include NFE2, Nrf1, and Nrf3. Nrf2 regulates the transcription of target genes by forming heterodimers with other bZIP proteins (such as MAF isoforms K/G/F) via the Neh domain (seven phylogenetically conserved functional domains) (Itoh et al., 1995). In the physiological state, Nrf2 is bound to KEAP 1 and labeled by ubiquitination, and rapidly degraded by the proteasome pathway (half-life 15–40 min), maintaining low basal activity (Cuadrado et al., 2019; Ulasov et al., 2022). Oxidative stress triggers the dissociation of the KEAP1-Nrf2 complex, which causes Nrf2 nuclear translocation and binding to antioxidant response elements (ARE). Activated Nrf2 regulates transcription of antioxidant enzymes and induces a series of antioxidant enzymes. The transcription of NADPH quinone oxidoreductase (NQO1), glutathione reductase (GR), glutathione S-transferase (GST) and other key antioxidant enzyme genes was activated (Zhang et al., 2021; Shi et al., 2022). In summary, Nrf2 controls the transcription of several antioxidant genes, thereby scavenging ROS and repairing oxidative damage, and plays a central protective role in chronic inflammatory diseases, such as RA (Hayes and Dinkova-Kostova, 2014). Animal models showed that Nrf2 deficient mice had significantly decreased oxidative phosphorylation (Holmström et al., 2013) and inhibited expression of antioxidant enzymes (such as CAT and γ-GCS), confirming the necessity of a regulatory network (Li et al., 2021). It is crucial to distinguish the disease-specific role of Nrf2 in RA from its systemic antioxidant effects. In the RA synovial microenvironment, Nrf2 serves as a metabolic and physical rheostat. Beyond simply quenching ROS, Nrf2 activation specifically modulates the metabolic reprogramming of fibroblast-like synoviocytes (FLS), shifting them from a pro-inflammatory glycolytic state back to oxidative phosphorylation (Rajendiran et al., 2022). Furthermore, Nrf2-mediated induction of HO-1 specifically antagonizes the RANKL-induced osteoclastogenesis, a process distinct from its detoxification roles in the liver (Maicas et al., 2011). This specific intervention in how inflammation drives bone loss makes Nrf2 a precise therapeutic target for RA structural damage.

Studies on juvenile idiopathic arthritis (JIA) have shown that Nrf2 activation in CD4^+^ T cells significantly reduces the levels of oxidative stress markers, remodels cellular metabolic characteristics, and inhibits IFNγ secretion (Rajendiran et al., 2022). In the RA model treated with dihydromyricetin (DMY), the expression of NQO1 in the synovial tissue was significantly upregulated, and the pro-inflammatory mediator COX-2 was significantly downregulated. Notably, the mRNA and protein expression levels of the Nrf2 downstream target genes HO-1 and NQO1 were characteristically decreased in RA synovium, which was effectively restored by DMY treatment, suggesting that this metabolite inhibits the expression of inflammatory cytokines by activating the Nrf2 pathway (Chu et al., 2018). In addition, resveratrol activates the Nrf2-ARE signaling pathway through the SIRT1/NF-κB/miR regulatory axis, which provides a new mechanism for ameliorating the pathological process of RA (Wang et al., 2020). Animal experiments have confirmed that Nrf2 knockout mice exhibit more severe joint damage and accumulation of oxidative stress markers in an antigen-induced arthritis (AIA) model, and the expression of antioxidant enzymes, such as HO-1 and γ-GCS, in articular cartilage is significantly impaired (Wruck et al., 2011). Notably, the incidence of arthritis was significantly increased in Nrf2-deficient mice, and the limb joint involvement was more severe, confirming that this pathway plays a key protective role in joint inflammation and degeneration (Maicas et al., 2011). Pharmacological studies have shown that sulfasalazine, a commonly used clinical drug, affects oxidative metabolism by inhibiting cystine/glutamate antitransporter (Gout et al., 2001). Based on current evidence, the targeted activation strategy of the Nrf2 signaling pathway has become a potential therapeutic direction for the intervention of RA and other diseases. By regulating the Nrf2 signaling pathway, it can simultaneously act on multiple pathological links, such as oxidative stress, inflammatory response, and cell metabolism, demonstrating the advantages of multi-target therapy. Therefore, activation of Nrf2 signaling pathway is considered to be a promising therapeutic strategy for the treatment of RA based on preclinical evidence. However, it is important to note that these findings are primarily derived from animal models and *in vitro* studies. Clinical translation has proven challenging. A phase 2 clinical trial (NCT00810836) evaluated dimethyl fumarate (DMF), an FDA-approved Nrf2 activator, in patients with active RA. Although DMF successfully activated the Nrf2 pathway and showed a downward trend in some inflammatory markers, it failed to achieve statistically significant improvements in predefined clinical outcome measures (unpublished data) (Kourakis et al., 2020). This gap between promising preclinical findings and clinical efficacy in human RA patients highlights the need for further investigation to optimize Nrf2-targeted therapeutic strategies.

### RAGE signaling

Advanced glycation end product receptor (RAGE) is a multiligand surface receptor, a transmembrane protein composed of an extracellular ligand binding domain, transmembrane region, and intracellular signaling domain, which can be present in both the transmembrane form (mediating signal transduction) and soluble form (sRAGE, antagonistic ligand) (Hudson and Lippman, 2018). Its multiligand binding properties include S100 protein family, HMGB1, β-amyloid, etc (Jangde et al., 2020). Especially under oxidative stress or high glucose environment. The binding of RAGE to AGEs (non-enzymatically glycated or oxidized proteins, lipids, and nucleic acids) activates downstream pro-oxidative and pro-inflammatory signals (Avalos et al., 2010; Luan et al., 2020), which further promotes ROS production.

In the pathological synovial environment of RA, RAGE is abnormally highly expressed in macrophages, T cells and synovial fibroblasts (Raucci et al., 2007; Rasheed and Haqqi, 2012). Binding of ligands (such as HMGB1 or S100B) to RAGE triggers the activation of MAPK (ERK1/2, JNK, p38) and NF-κB pathways, drives the release of TNF-α, IL-6 and other pro-inflammatory factors, and promotes matrix metalloproteinase (MMPs) -mediated bone erosion (Sorci et al., 2013; Riehl et al., 2009). Clinical evidence shows that the levels of RAGE ligands (such as HMGB1) in the synovial fluid and serum of patients with RA are significantly increased, and the level of sRAGE is negatively correlated with disease activity (Pullerits et al., 2005; Chen et al., 2009). In addition, exogenous sRAGE competitively binds ligands, inhibits the secretion of TNF-α by synovial macrophages, and attenuates bone destruction in a mouse model of collagen-induced arthritis (Sunahori et al., 2006). In addition, anti-HMGB1 antibody was found to block HMGB1-RAGE interaction and reduce joint inflammation (Park et al., 2015). Targeting the intracellular signaling domain of RAGE (e.g., blocking the Cdc42/Rac pathway) can inhibit the inflammatory cascade (Raucci et al., 2007; Riehl et al., 2009; Nienhuis et al., 2009), and some scholars have suggested that RAGE is related to RA through its ability to amplify inflammatory pathways. These strategies provide novel molecular targets for RA treatment by precisely regulating RAGE-ligand interactions. However, it is crucial to note that all therapeutic evidence supporting RAGE inhibition for RA is derived exclusively from preclinical studies, including animal models (e.g., collagen-induced arthritis, IL-1Ra knockout mice) and *in vitro* experiments. To date, no RAGE inhibitors have been evaluated in clinical trials for RA patients. While human observational studies have demonstrated that serum sRAGE levels are decreased in RA patients compared to healthy controls (872 ± 65 pg/mL vs. 1,290 ± 78 pg/mL) and negatively correlate with disease activity (Pullerits et al., 2005; Chen et al., 2009), these are purely biomarker studies without therapeutic intervention. The most clinically advanced RAGE inhibitor, azeliragon (TTP488), underwent Phase 3 clinical trials for Alzheimer’s disease (STEADFAST trial, NCT02080364) but was terminated in 2018 due to failure to meet primary efficacy endpoints (Burstein et al., 2018). Notably, the high-dose arm was discontinued early due to safety concerns including increased falls and confusion. The translation of RAGE-targeting strategies from promising preclinical RA models to clinical application remains a significant unmet challenge.

### NOX signaling

The NADPH oxidase family is a key enzyme system for the generation of reactive oxygen species, and its members participate in the body’s defense by catalyzing the conversion of molecular oxygen to superoxide and hydrogen peroxide. The NADPH oxidase family was first discovered in the membrane of phagocytes, and its function is to achieve pathogen clearance by producing reactive oxygen species (ROS). Seven isoforms (NOX1-5 and DUOX1/2) of the enzyme family have been identified, which are characterized by the transmembrane flavocytochrome complex b558 (catalytic core), consisting of gp91phox and p22phox subunits. In addition to the cytoplasmic regulatory subunits, p47phox, p67phox, p40phox, and gtpase Rac (Dong et al., 2011; Vermot et al., 2021; Eid et al., 2022).

Recent studies have shown that this enzyme family has important pathological significance in cardiovascular diseases, autoimmune diseases and inflammatory response (Vendrov et al., 2015; Piera-Velazquez and Jimenez, 2015). Among these, NOX4 is the main contributor to ROS production (Drevet et al., 2018) and plays a key role in the pathogenesis of RA. Experimental data have shown that NOX4-mediated superoxide release was significantly enhanced by TNF-α stimulation in RA synoviocytes (Chenevier-Gobeaux et al., 2006). Further studies have shown that the migration and invasion of fibroblast-like synoviocytes (FLS) from RA patients are regulated by ROS levels, and NOX4 promotes this pathological process by upregulating the expression of VCAM1 and VEGF. Notably, cellular experiments comparing seven RA patients with five osteoarthritis patients showed that the NADPH oxidase-specific regulatory mechanism exhibited constitutive activation and enhanced cytokine induction in RA synoviocytes (Chenevier-Gobeaux et al., 2006). Based on this mechanism, the research team proposed that NOX4 may be a novel molecular target for RA treatment. This study provides a theoretical basis for the development of novel targeted therapeutic strategies.

### NF-κB signaling

Excess reactive oxygen species (ROS) are able to activate nuclear factor-κB (NF-κB), a redox-sensitive protein complex that plays a central role in inflammatory responses. NF-κB activation promotes the transcription and release of pro-inflammatory mediators such as interleukin-6 (IL-6) and tumor necrosis factor-α (TNF-α) (Xu et al., 2023; Capece et al., 2022). Several studies have demonstrated that suppressing NF-κB signaling leads to a marked reduction in inflammation, oxidative stress, fibrosis, hypertrophy, and programmed cell death in both cellular and animal models (Xu et al., 2023; Li et al., 2019). In addition, the NF-κB signaling pathway can act synergistically with advanced glycation end products (AGEs) and NADPH oxidase (NOX) signaling pathways, causing more severe oxidative damage (Tang et al., 2018; Zou et al., 2019). However, the specific relationship between these signaling pathways requires further study.

In RA, the NF-κB signaling pathway plays a central transcriptional role (Wang et al., 2018). In macrophages, the activation of NF-κB is commonly induced by ligands of Toll-like receptors (TLRs), including lipopolysaccharide (LPS), which subsequently promotes the transcription of multiple cytokines, chemokines, and other mediators of inflammation (Wang et al., 2018; Fearon et al., 2016; Huang et al., 2007). Previous studies have shown that TLR4 is significantly activated in patients (Huang et al., 2007). The activation of NF-κB is mainly regulated through two different pathways, the “classical” and “alternative” pathways (Wang et al., 2018). In the classical signaling cascade, potent activators like LPS, interferon-gamma (IFN-γ), and tumor necrosis factor-alpha (TNF-α) trigger the phosphorylation of IκB kinase (IKK),this activation results in the serine phosphorylation and subsequent breakdown of IκBα, consequently, NF-κB is liberated, migrates into the nucleus, and modulates the expression of various genes, such as those encoding TNF-α, IL-1β, IL-6, and inducible nitric oxide synthase (iNOS) (Wang et al., 2018; Fearon et al., 2016; Huang et al., 2007; Min et al., 2013). On the other hand, the alternative pathway is activated by specific members of the tumor necrosis factor receptor (TNFR) superfamily, such as CD40, lymphotoxin-β receptor and receptor activator of NF-κB (RANK). This pathway relies on NF-κB-inducing kinase (NIK, also known as MAP3K14) and IKKα, which facilitate the processing of p100 to p52, culminating in the formation of the p52/RelB heterodimer and activation of downstream signaling (Jimi and Ghosh, 2005; Hayden and Ghosh, 2014). The classical NF-κB pathway not only regulates the expression of inflammatory cytokines but is also continuously activated by these cytokines; therefore, it is considered a potential target for the treatment of RA (Simmonds and Foxwell, 2008; Sun, 2017).

In the study of RA, activated NF-κB transcription factors have been demonstrated in a variety of experimental models, including cultured synovial fibroblasts (Fujisawa et al., 1996; Roshak et al., 1996; Yamasaki et al., 2001), human arthritic tissues (Yamasaki et al., 2001) and experimentally induced RA animal models (Han et al., 1998; Tsao et al., 1997). In addition, constitutive activation of the classical NF-κB pathway has been frequently observed in the synovial tissues of patients (Asahara et al., 1995; Gilston et al., 1997; Miyazawa et al., 1998). Activation of the classical NF-κB pathway has also been demonstrated in a mouse model of collagen-induced arthritis (Han et al., 1998). Therefore, inhibitors of NF-κB activation are potential drugs for the treatment of RA. Indeed, biotherapies that directly target NF-κB-driven gene products, such as TNF-α, IL-6, and IL-1, have shown remarkable success in clinical trials, further demonstrating the importance of this pathway in RA treatment (Sacre et al., 2005).

The oxidative stress pathways in RA function as an integrated network where RAGE, NOX, NF-κB, and Nrf2 converge and amplify each other. NF-κB serves as the central hub: RAGE activation (via AGEs/HMGB1 binding) triggers MAPK cascades that activate NF-κB (Sorci et al., 2013; Riehl et al., 2009), while NOX-derived ROS directly oxidize IKK subunits to enhance NF-κB activation (Capece et al., 2022). Once activated, NF-κB upregulates both RAGE and NOX4 expression, creating positive feedback loops (Raucci et al., 2007; Drevet et al., 2018). Additionally, RAGE and NOX exhibit cross-amplification: AGE-RAGE signaling stimulates NOX activity, while NOX-derived ROS accelerates AGE formation (Avalos et al., 2010; Riehl et al., 2009).

The Nrf2 pathway normally counterbalances this network by inducing antioxidant enzymes (HO-1, NQO1, GST) and competing with NF-κB for transcriptional resources (Zhang et al., 2021; Hayes and Dinkova-Kostova, 2014). However, Nrf2 is characteristically suppressed in RA synovium (Chu et al., 2018), removing this protective brake and allowing unchecked amplification of oxidative-inflammatory signals. This integrated framework explains why single-pathway inhibition may be insufficient and supports multi-targeted therapeutic approaches that simultaneously activate Nrf2 while inhibiting NF-κB, RAGE, and NOX.

### Role of OA, an antioxidant, on rheumatoid arthritis

**FIGURE 2:**
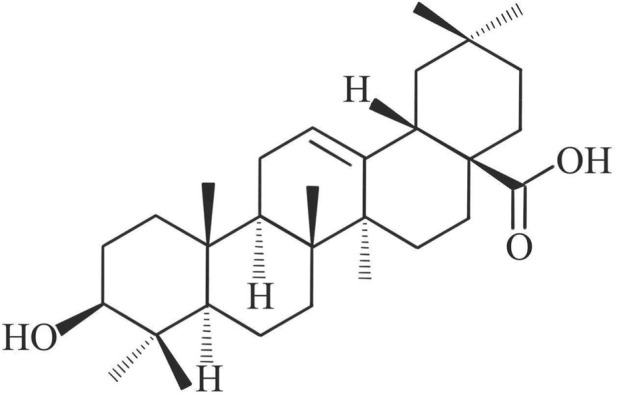
Chemical structure of the investigational agent oleanolic acid (OA). This pentacyclic triterpenoid exhibits significant dual anti-inflammatory and antioxidant properties targeted at RA pathology.

There is consensus on the key role of oxidative stress regulatory pathways in the pathological mechanism of RA. Oxidative stress plays a significant role in RA treatment. Current research indicates that numerous natural metabolites can alleviate the development of RA by mitigating oxidative stress (Xu et al., 2023; Islam et al., 2016). Although research utilizing animal and cellular models has documented the efficacy of certain natural metabolites against RA, their application in human clinical trials remains unexplored (Xu et al., 2023). Oleanolic acid (OA; 3β-hydroxy-olea-12-en-28-oic acid) is a pentacyclic triterpenoid ubiquitously distributed throughout the plant kingdom, existing either as a free acid or as an aglycone for triterpenoid saponins. Historically, the primary commercial and research-standard source of OA is the olive plant, *Olea europaea* L. (Oleaceae), particularly concentrated in the fruit and leaf cuticular waxes. Other significant sources include the marigold herb (*Calendula officinalis* L.; Asteraceae), whose wild-type and hairy roots produce OA and its saponins, and the mistletoe herb (*Viscum album* L.; Santalaceae). However, it is critical to note that the pharmacological efficacy reported in the literature often varies significantly depending on the extraction solvent (e.g., ethanol vs. aqueous extracts) and the specific plant organ utilized, a factor that necessitates more rigorous standardization in preclinical RA models. Given the integrated nature of oxidative stress pathways in RA, therapeutic agents that modulate multiple nodes may offer superior efficacy. OA demonstrates such multi-targeted properties by simultaneously: activating Nrf2 to enhance antioxidant defenses; suppressing RAGE ligands (HMGB1, AGEs); inhibiting NOX activity; and blocking NF-κB through IKKβ inhibition and prevention of nuclear translocation. This positions OA as a promising candidate for breaking the self-reinforcing oxidative-inflammatory cycle in RA (Figure 2).

In terms of the regulation of the Nrf2 signaling pathway, several studies have shown that OA activates the pathway; for example, OA directly binds to the Keap1 protein to block the ubiquitination and degradation of Nrf2; Activation of kinase cascades such as ERK1/Akt and AMPK promotes Nrf2 nuclear translocation (Fernández-Aparicio et al., 2022). This effect can significantly enhance the expression of antioxidant enzymes, and the experimental results showed that the hepatoprotective effect of OA was significantly reduced after Nrf2 gene knockout (Liu et al., 2023). OA activates Nrf2, which not only exerts a broad-spectrum antioxidant effect but, more importantly, specifically inhibits the invasive behavior of RA-FLS. Through the Nrf2 pathway, OA can downregulate the expression of matrix metalloproteinases in synovial tissue, thereby directly protecting articular cartilage from erosion. This regulation of synovial tissue invasiveness is a disease-specific manifestation of OA in the treatment of RA. Another report found that OA activates Nrf2 and reduces oxidative stress levels while OA is continuously depleted (Ouyang et al., 2023). More importantly, one study suggested that OA promotes the nuclear accumulation of Nrf2, leading to the induction of NrF2-dependent genes (Reisman et al., 2009). In conclusion, OA can activate the Nrf2 signaling pathway, but the exact mechanism remains unclear. More importantly, similar to other Nrf2 activators, OA has demonstrated anti-arthritic effects exclusively in preclinical models. To date, no clinical trials have been conducted to evaluate the efficacy of OA specifically for RA treatment in humans. The failed or inconclusive clinical trials of other Nrf2 activators in RA underscore the challenges in translating preclinical efficacy into clinical benefit.

In a vascular endothelial cell inflammation model, OA significantly inhibited the extracellular secretion of HMGB1 (Yang et al., 2012), and this effect was also validated in serological testing of rats with subarachnoid hemorrhage. It is worth noting that studies on animal models of diabetes have shown that OA inhibits the activities of aldose reductase (AR) and sorbitol dehydrogenase through targeting (Jang et al., 2010). Given the close association between the sorbitol fructose metabolic axis and the generation of advanced glycation end products (AGEs), the dual inhibitory effect of OA on these two enzymes can significantly reduce the accumulation of tissue AGEs (Castellano et al., 2013). Because of the specific binding of HMGB1 and AGEs to RAGE receptors to activate downstream signals, OA may regulate the oxidative stress response pathway by intervening in the key link in RAGE ligand generation. However, all evidence supporting OA’s effects on the RAGE pathway comes from preclinical models, with no clinical validation in RA patients. Human studies have been limited to biomarker observations—sRAGE levels inversely correlate with disease activity—but no interventional trials have tested whether blocking RAGE improves RA outcomes (Chen et al., 2009). The failed clinical development of azeliragon, a potent RAGE antagonist, in Alzheimer’s diseasehighlights the difficulty in translating preclinical RAGE inhibition into clinical benefit for OA and relate metabolites (Burstein et al., 2018).

Recent studies have found that OA can specifically inhibit the activity of NADPH oxidase, and animal experiments have shown that mice exposed to PCB had significantly reduced serum MDA levels after OA intervention, along with the suppression of the expression of NOX 4 and its associated regulatory genes (Su et al., 2018). This suggests that NADPH oxidase may be the main target of ROS scavenging by OA (Fernández-Aparicio et al., 2022). At the same time, the core enzyme composition of NADPH oxidase includes P47 and P67, and experimental data has shown that five triterpenoid saponins of OA isolated from the roots and stems of sea anemones can inhibit the production of superoxide by inhibiting P47 and P67 (Wei et al., 2012).

**FIGURE 3:**
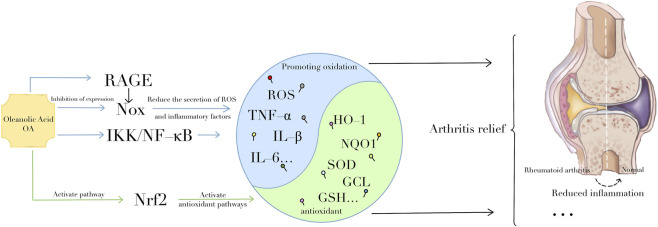
The role of OA in rheumatoid arthritis. OA can inhibit the activation of RAGE, NOX, and NF-κB pathways, promote the Nrf2 signaling pathway. By altering the expression of these signaling pathways, OA can inhibit oxidative stress in many ways, thereby alleviating rheumatoid arthritis.

Similar to the Nrf2 signaling pathway, research on the regulatory mechanism of the NF-κB signaling pathway made a breakthrough discovery: OA not only inhibited IKK β activity (Fernández-Aparicio et al., 2022) but also dual blocked NF-κB nuclear translocation by downregulating MafK-mediated p65 acetylation (Hwang et al., 2014). In an insulin resistance model, OA upregulated the expression of IRS1/GLUT4 and decreased the levels of pro-inflammatory cytokines IL-6 and TNF-α through this pathway (Li et al., 2015). Notably, ROS-induced NF-κB activation has a positive feedback relationship with PTP1B overexpression (Panzhinskiy et al., 2013) and inhibition of PTP1B by OA may be an important link in its antioxidant network (Liu Q. C. et al., 2013; Liu Q. et al., 2014) (Figure 3).

Although a large number of preclinical studies have confirmed the antioxidant potential of OA (Ayeleso et al., 2017), this has not advanced to the clinical trial stage. This clinical gap is consistent with the broader field of antioxidant therapy. For instance, while specific agents like Edaravone for acute ischemic stroke and MitoQ for vascular function have shown promise, most large-scale trials for chronic diseases (e.g., Parkinson’s and cardiovascular prevention) have failed to demonstrate long-term clinical efficacy (Sies and Jones, 2020). This suggests that in humans, ROS may serve more reliably as a biomarker of pathological state rather than a primary therapeutic target, unless interventions can achieve high spatiotemporal specificity. The complexity of its mechanism of action is reflected in the following aspects: multi-pathway coordination; however, the primary and secondary relationships are not clear, and there are differences in signal transduction in different tissues. The dose-response relationship needs to be systematically studied. The solutions to these scientific problems will lay the foundation for clinical translation. At the same time, a critical and balanced assessment of the literature reveals a significant mechanistic paradox regarding the redox-modulating properties of OA. While the prevailing narrative in RA research focuses on its antioxidant and Nrf2-mediated cytoprotective effects, evidence from oncological models presents a starkly contradictory pro-oxidant role. Specifically, research on human pancreatic cancer cells (Wei et al., 2013) demonstrates that OA treatment leads to a substantial induction of intracellular ROS levels, which serves as the primary trigger for mitochondrial-mediated apoptosis rather than suppressing oxidative damage. Similarly, in hepatocellular carcinoma models OA (and its derivatives) has been shown to activate the AMPK/mTOR signaling pathway via a ROS-mediated mechanism, promoting autophagy-dependent cell death (Liu J. et al., 2014). These findings suggest that OA’s role is highly environment-dependent: it neutralizes ROS in the inflammatory setting of RA,however, in malignant or highly stressed metabolic states, it switches to a pro-oxidant agent. This context-dependent duality constitutes a critical knowledge gap—current RA-focused studies largely ignore the potential for OA to inadvertently induce oxidative stress in non-target tissues or under specific pathological fluctuations. Recognizing this contradiction is essential for a rigorous evaluation of OA’s safety profile and therapeutic window, as the assumption of its “universal antioxidant” nature is clearly challenged by high-quality mechanistic data in adjacent fields.

## Lessons from previous antioxidant therapies and potential of oleanolic acid

Despite the established role of oxidative stress in RA pathogenesis, traditional antioxidant supplements have failed to demonstrate significant clinical efficacy. A systematic review of 20 randomized controlled trials (11 in inflammatory arthritis and nine in osteoarthritis) found no convincing evidence that selenium, vitamins A, C, or E are effective in treating arthritis (Canter et al., 2007). Recent systematic reviews and meta-analyses of N-acetylcysteine (NAC) also showed that despite its antioxidant and anti-inflammatory properties, its efficacy as adjuvant therapy for RA remains controversial (He et al., 2024; Esalatmanesh et al., 2022). While selenium supplementation showed some benefits for joint pain, its effects on erythrocyte sedimentation rate, C-reactive protein levels, morning stiffness, and joint swelling remain unclear and require further clinical trials (Zamani et al., 2024). These failures can be attributed to several key factors. A primary drawback is their limited single-target potency. Traditional antioxidants (e.g., vitamins C, E) function primarily as direct ROS scavengers without modulating enzymatic sources of ROS (such as NADPH oxidase) or activating endogenous antioxidant defense systems. Furthermore, their clinical utility is often hampered by insufficient tissue penetration and poor bioavailability. Although vitamin E can distribute into blood and synovial fluid, its concentration in the joint space may be insufficient to counteract intense oxidative stress (Sutipornpalangkul et al., 2009). A critical shortcoming of traditional antioxidants is their inherent lack of direct anti-inflammatory activity. Simple antioxidants do not directly suppress the pro-inflammatory cytokine cascade (TNF-α, IL-6, IL-1β) that drives RA pathology. Since oxidative stress and inflammation form a self-perpetuating cycle in RA, single interventions are unlikely to break this vicious cycle.

But OA represents a fundamentally different therapeutic strategy from traditional antioxidants. First, OA can be coordinated with multiple channels (Nrf2, NOX4, RAGE, NF-κB). OA addresses the complex immunopathology of RA by suppressing TNF-α and IL-6 production, reducing synoviocyte migration and invasion, and modulating immune cell function (Li et al., 2015) to show integrated anti-inflammatory and immunomodulatory effects. Furthermore, OA has demonstrated excellent safety in various experimental models and is naturally present in the human diet, supporting chronic administration (Bachhav et al., 2011; Martínez-González et al., 2008; Sapkota and Choi, 2022). However, rigorous clinical investigation is necessary to determine whether these theoretical advantages translate into meaningful therapeutic benefits for RA patients.

## Recent advances in combination therapies and synergistic effects of OA

Despite its significant pharmacological potential, the clinical application of OA as a monotherapy is often constrained by its poor aqueous solubility and low oral bioavailability. To overcome these limitations, recent research (2023–2025) has shifted towards exploring OA in synergistic combinations with other bioactive metabolites, aiming to achieve a “multi-target, multi-pathway” therapeutic effect (Shaharyar et al., 2025). Within a clinical framework, the 2024 Japan College of Rheumatology (JCR) guidelines emphasize the continuous evolution of combination strategies (e.g., MTX with biologics), creating a potential niche for OA as a natural adjunct to mitigate drug-induced oxidative stress and enhance overall efficacy (Nakayama et al., 2024). Innovative chemical engineering has further validated the synergistic potential of OA through the development of hybrid molecules and advanced delivery systems. In 2023, researchers developed novel Curcumin-OA hybrid metabolites using a succinate linker, which demonstrated superior pharmacological profiles compared to individual metabolites by simultaneously targeting multiple inflammatory cascades (Sowa-Kasprzak et al., 2023). Furthermore, breakthrough studies in 2024 and 2025 have pioneered co-assembled nanoparticles, such as the OLA-Cur system, which utilizes the structural properties of OA to enhance the solubility and stability of curcumin, leading to improved cartilage protection and ROS scavenging in joint disease model (Liu et al., 2025; Zhang et al., 2025). Notably, OA’s role has expanded beyond an active metabolite to a structural stabilizer; it has been integrated into the lipid bilayers of liposomes carrying SGLT2 inhibitors and curcumin to enhance membrane integrity and prolong drug release (Profire et al., 2025).

While many combinations show promise, recent 2025 comprehensive reviews have noted that not all natural product pairings result in synergy. Certain combinations may exhibit antagonistic interactions or inconsistent efficacy depending on the specific pathological microenvironment and dosage ratios (Han et al., 2025). This highlights the necessity for precise formulation optimization in future OA-based integrated therapies.

## Conclusions and perspectives

Advances in the molecular mechanisms of rheumatoid arthritis (ROS) have indicated that the oxidative stress regulatory network plays a central role in the disease process. A substantial body of preclinical evidence demonstrates that joint pathological damage can be significantly improved by regulating the oxidative activity of the NF-κB/NOX/RAGE signaling axis or activating the antioxidant mechanism of the Nrf2 pathway in animal models and cellular systems. However, clinical validation of these pathway-specific interventions in human RA patients remains largely absent. Critically, clinical trials evaluating Nrf2 activators (dimethyl fumarate) failed to demonstrate significant clinical benefit in RA patients despite successful pathway activation (NCT00810836, unpublished data), and no RAGE or NOX inhibitors have advanced to RA clinical trials. While human biomarker studies confirm altered pathway activity in RA patients*—*such as decreased sRAGE levels (Pullerits et al., 2005; Chen et al., 2009)—observational correlations do not establish therapeutic efficacy. Furthermore, the mechanisms underlying the co-regulation of these key pathways and their spatiotemporal specificity remain unexplored, which metabolites the difficulty in developing precise therapeutic strategies. This substantial gap between promising preclinical findings and clinical translation must be acknowledged and addressed through well-designed clinical trials before oxidative stress-targeting strategies can be recommended for RA treatment. The skepticism regarding ROS-targeted therapy stems from the failure of global antioxidant supplementation in clinical trials. Direct evidence for ROS regulation altering disease progression in humans is scarce. Future research on metabolites like OA must therefore shift from general ROS removal to targeted control. This entails targeting specific enzymatic sources of ROS (like NOX4 in FLS) at specific stages of synovitis, rather than non-specific neutralization. Only by addressing this spatiotemporal complexity can we determine if ROS regulation is a viable disease-modifying strategy or merely a tool for disease monitoring.

The current clinical treatment of RA mainly involves non-steroidal anti-inflammatory drugs (NSAIDs), disease-modifying antirheumatic drugs (DMARDs), targeted biological agents, and other immunomodulatory methods. However, basic studies have shown that an abnormal increase in ROS levels can be detected during the initial stages of synovitis. This oxidative stress microenvironment not only stimulates the abnormal proliferation of synovial fibroblasts but also accelerates the degradation of cartilage matrix by inducing the cascade release of TNF-α, IL-6, and other pro-inflammatory factors. This finding suggests that early intervention against oxidative stress may be a key factor in delaying disease progression. OA, a pentacyclic triterpenoid, exhibits multidimensional biological effects owing to its unique molecular structure. OA may exert its antioxidant effect by quenching ROS, inhibiting lipid peroxidation, or indirectly stimulating cellular antioxidant defenses (Stadtman and Levine, 2000). In addition, a large portion of the ongoing research has focused on natural products, suggesting that their therapeutic value deserves more attention. They also possess various biological activities, including anti-inflammatory, antioxidant, immunomodulatory, antibacterial, and antiviral effects. Various experimental models have also confirmed that OA is safe and beneficial to the human body (Bachhav et al., 2011; Martínez-González et al., 2008; Sapkota and Choi, 2022). Notably, its immunomodulatory properties and anti-inflammatory activity make it a unique advantage in the field of RA treatment. Consequently, OA shows promise as a novel therapeutic approach for RA. Nonetheless, further investigation into its mechanism of action, target specificity, bioavailability, and potential interactions with other drugs is necessary before it can be applied in clinical practice. Based on OA’s multi-target antioxidant and anti-inflammatory mechanisms, its potential as adjuvant therapy for RA warrants discussion. Theoretically, OA may produce synergistic effects with conventional DMARDs (such as methotrexate) or biologics (such as TNF-α inhibitors). However, no clinical trial data currently exist for OA combination therapy with these RA standard treatments. From a mechanistic perspective, OA may have complementary actions with existing RA therapies: OA may demonstrate potential synergy with methotrexate: Methotrexate exerts anti-inflammatory effects by inhibiting dihydrofolate reductase while potentially inducing oxidative stress and hepatotoxicity. Animal studies show that OA at low doses has hepatoprotective effects, protecting mice from liver injury induced by combination antitubercular drugs (rifampicin, isoniazid, pyrazinamide) (Gutiérrez-Rebolledo et al., 2016). This study found that an OA/ursolic acid mixture (100–200 μg/mouse/day, subcutaneous injection for 11 weeks) significantly decreased serum transaminase levels and ameliorated histopathological alterations. This suggests OA may mitigate methotrexate-related hepatotoxicity while providing additional antioxidant protection through Nrf2 pathway activation. OA may also show potential synergy with biologics: OA can inhibit production of pro-inflammatory cytokines including TNF-α and IL-6, with mechanisms partially overlapping but distinct from TNF-α inhibitors (such as infliximab, adalimumab) (Bha et al., 2014). Biologics directly neutralize cytokines, while OA regulates inflammatory responses at the transcriptional level through NF-κB pathway suppression and Nrf2 pathway activation. This mechanistic difference may produce synergistic anti-inflammatory effects and potentially reduce the required doses of biologics. Theoretically, OA has potential as RA adjuvant therapy, possibly producing synergistic anti-inflammatory effects with methotrexate or biologics while mitigating drug toxicity. However, clinical data supporting its safety and efficacy are currently lacking. OA’s dose-dependent hepatotoxicity and effects on hepatic metabolic enzymes are major safety considerations. Studies show that OA at doses ≥90 mg/kg for five consecutive days can induce liver injury, characterized by elevated serum bile acids and cholestasis (Liu J. et al., 2013) and other studies show that OA treatment decreases CYP2E1 activity (Jeong, 1999) and various CYP450 enzyme activities (Liu et al., 1995), potentially affecting clearance of hepatically metabolized drugs Systematic drug interaction studies, dose optimization, and safety assessments are needed before human clinical trials. The *in vitro* and *in vivo* experimental evidence for OA across pathways discussed above, including study models, concentrations, molecular targets, and key findings, is comprehensively summarized in Table 1.

**TABLE 1:** Summary of oleanolic acid (OA) studies in rheumatoid arthritis: study type, experimental conditions, concentration, and targets.

Study type	Model/Cell line	Concentration/Dose	Target/Pathway	Key findings	Source/Extract	References
*In vitro* studies						
*In vitro*	HUVECs (LPS-induced HMGB1 inflammation model)	Increasing concentrations tested (range not fully disclosed); LPS stimulus: 100 ng/mL	RAGE/HMGB1/NF-κB/TNF-α	Inhibited LPS-mediated HMGB1 release; downregulated HMGB1-dependent monocyte adhesion/migration; suppressed NF-κB activation and TNF-α production	Isolated compound (purchased from Sigma)	Yang et al. (2012)
*In vitro*	RAW 264.7 macrophages (LPS-induced, 1 μg/mL)	10 μM (primary functional dose); cytotoxicity range tested: 10, 25, 50 μM	NF-κB/MafK/Nrf2	Inhibited LPS-induced NO and PGE2 production; suppressed NF-κB via decreased MafK expression and MafK-mediated p65 acetylation; activated Nrf2	Isolated compound (Prunella vulgaris extract-derived, Sigma)	Hwang et al. (2014)
*In vitro*	Insulin-resistant HepG2 cells (200 μmol/L sodium oleate induction)	5, 10, and 25 μmol/L OA (24 h); effective dose: 10–25 μmol/L	NF-κB/IRS1-GLUT4 axis	Upregulated IRS1 and GLUT4 protein expression; decreased IL-6 and TNF-α; reduced NF-κB protein expression	Isolated compound (purchased from National Institutes for Food and Drug Control, Beijing)	Li et al. (2015)
*In vitro*	Human pancreatic cancer cells	Dose-dependent; cytotoxic range used (exact μM not reported in review text)	ROS/mitochondria/lysosomal membrane	Induced intracellular ROS; caused mitochondrial depolarization and lysosomal membrane permeabilization; triggered apoptosis (pro-oxidant role in cancer context)	Isolated compound	Wei et al. (2013)
*In vitro*	Hepatocellular carcinoma cells	Dose-dependent (exact μM not reported in review text)	AMPK/mTOR/ROS	Activated AMPK/mTOR via ROS-mediated mechanism; promoted autophagy-dependent cell death	Isolated compound (OA + derivatives)	Liu J. et al. (2014)
*In vitro*	Human neutrophils (cell-free superoxide assay)	Not specified (inhibitory concentrations of individual saponins)	NADPH oxidase (P47/P67 subunits)	Inhibited stimulus-induced superoxide generation by blocking P47 and P67 translocation/phosphorylation	Isolated saponins from sea anemone (Anemone raddeana) rhizome	Wei et al. (2012)
*In vivo* studies						
*In vivo*	Rats with subarachnoid hemorrhage	Dose used in original study (not specified in review); serological validation performed	RAGE/HMGB1 pathway	Reduced serum HMGB1 levels post-hemorrhage; confirmed the endothelial anti-HMGB1 effect of OA *in vivo*	Isolated compound	Yang et al. (2012)
*In vivo*	C57BL/6 mice exposed to Aroclor 1254 (PCBs mixture)	Not precisely specified in abstract; *in vivo* gavage model; OA dose range in similar PCB studies: 20–50 mg/kg	NOX4/HNF1b/PPARγ/redox signaling	Significantly reduced serum MDA; suppressed NOX4 and associated regulatory gene expression; attenuated adiposity and insulin resistance via HNF1b-mediated redox regulation	Isolated compound	Su et al. (2018)
*In vivo*	Streptozotocin-induced diabetic mice/*in vitro* enzymatic assay	Not specified; enzymatic inhibitory studies (IC50 not reported for OA in this context)	Aldose reductase/sorbitol dehydrogenase (AGE/RAGE upstream)	Inhibited aldose reductase and sorbitol dehydrogenase activities; reduced tissue AGE accumulation by suppressing polyol pathway flux	Isolated compound	Jang et al. (2010)
*In vivo*	Mice with antitubercular drug-induced liver injury (rifampicin + isoniazid + pyrazinamide)	100–200 μg/mouse/day, subcutaneous injection for 11 weeks	Hepatoprotection/Nrf2 pathway	Significantly decreased serum transaminase (ALT/AST) levels; ameliorated liver histopathology; hepatoprotective—relevant to potential MTX toxicity mitigation	Isolated compounds (OA/ursolic acid mixture, equal ratio)	Gutiérrez-Rebolledo et al. (2016)
*In vivo*	Wild-type and Nrf2-null C57BL/6 mice (acetaminophen hepatotoxicity model)	90 mg/kg i.p., once daily for 3 days (Nrf2 activation dose)	Nrf2-Keap1/Nqo1/HO-1/Gclc/metallothionein	Increased Nrf2 nuclear accumulation (+432%) and target gene induction (Nqo1 enzyme activity +154%) in WT only; hepatoprotective effect significantly reduced in Nrf2-null mice — confirmed Nrf2 dependency	Isolated compound	Reisman et al. (2009)
*In vivo*	C57BL/6 mice and rats (hepatotoxicity safety model)	≥90 mg/kg for 5 consecutive days (toxic threshold); hepatoprotective dose: 25–100 mg/kg s.c. for 3 days	Bile acid metabolism/cholestasis	Doses ≥90 mg/kg/5 days induced liver injury, elevated serum bile acids, and cholestasis — critical safety threshold for preclinical-to-clinical translation	Isolated compound	Liu J. et al. (2013)
*In vivo*	Mice (CYP450 modulation study)	100 and 200 μmol/kg s.c. for 3 days	CYP2E1/CYP450 enzymes (drug metabolism safety)	Dose-dependent reduction of liver CYP450 (25%–37%) and cytochrome b5 (15%–21%); decreased CYP2E1, coumarin 7-hydroxylase, ethoxyresorufin O-dealkylase activities—drug interaction concern	Isolated compound	Liu et al. (1995)

Abbreviations: OA, oleanolic acid; RA, rheumatoid arthritis; FLS, fibroblast-like synoviocytes; HMGB1, high-mobility group box 1; AGEs, advanced glycation end products; RAGE, receptor for advanced glycation end products; NOX4, NADPH, oxidase 4; MDA, malondialdehyde; MMPs, matrix metalloproteinase; IKKβ, IκB kinase beta; Nrf2, nuclear factor erythroid 2-related factor 2; PTP1B, protein tyrosine phosphatase 1B; ROS, reactive oxygen species; PCB, polychlorinated biphenyls; MTX, methotrexate. Note: “Not specified” in concentration/dose column indicates the original study did not report a specific concentration or that the reviewer could not retrieve exact doses from available citations. Studies marked as isolated compound used pure OA, rather than a plant extract.
